# Lymphocyte-Infiltrated Periportal Region Detection With Structurally-Refined Deep Portal Segmentation and Heterogeneous Infiltration Features

**DOI:** 10.1109/OJEMB.2024.3379479

**Published:** 2024-03-20

**Authors:** Hung-Wen Tsai, Chien-Yu Chiou, Wei-Jong Yang, Tsan-An Hsieh, Cheng-Yi Chen, Che-Wei Hsu, Yih-Jyh Lin, Min-En Hsieh, Matthew M. Yeh, Chin-Chun Chen, Meng-Ru Shen, Pau-Choo Chung

**Affiliations:** Department of Pathology, National Cheng Kung University Hospital, College of MedicineNational Cheng Kung University 34912 Tainan 701 Taiwan; Department of Electrical EngineeringNational Cheng Kung University 34912 Tainan 701 Taiwan; Department of Artificial Intelligence and Computer EngineeringNational Chin-Yi University of Technology 63372 Taichung 411030 Taiwan; Institute of Computer and Communication EngineeringNational Cheng Kung University 34912 Tainan 701 Taiwan; Department of Cell Biology and AnatomyCollege of MedicineNational Cheng Kung University 34912 Tainan 701 Taiwan; Department of Surgery, National Cheng Kung University Hospital, College of MedicineNational Cheng Kung University 34912 Tainan 701 Taiwan; Department of Laboratory Medicine and PathologyUniversity of Washington School of Medicine 12353 Seattle WA 98195 USA; Department of StatisticsNational Cheng Kung University 34912 Tainan 701 Taiwan; Department of Pharmacology, National Cheng Kung University Hospital, College of MedicineNational Cheng Kung University 34912 Tainan 701 Taiwan

**Keywords:** Deep learning, heterogeneous features, interface hepatitis, Ishak grading, structural analysis

## Abstract

*Goal*: The early diagnosis and treatment of hepatitis is essential to reduce hepatitis-related liver function deterioration and mortality. One component of the widely-used Ishak grading system for the grading of periportal interface hepatitis is based on the percentage of portal borders infiltrated by lymphocytes. Thus, the accurate detection of lymphocyte-infiltrated periportal regions is critical in the diagnosis of hepatitis. However, the infiltrating lymphocytes usually result in the formation of ambiguous and highly-irregular portal boundaries, and thus identifying the infiltrated portal boundary regions precisely using automated methods is challenging. This study aims to develop a deep-learning-based automatic detection framework to assist diagnosis. *Methods*: The present study proposes a framework consisting of a Structurally-REfined Deep Portal Segmentation module and an Infiltrated Periportal Region Detection module based on heterogeneous infiltration features to accurately identify the infiltrated periportal regions in liver Whole Slide Images. *Results*: The proposed method achieves 0.725 in F1-score of lymphocyte-infiltrated periportal region detection. Moreover, the statistics of the ratio of the detected infiltrated portal boundary have high correlation to the Ishak grade (Spearman's correlations more than 0.87 with p-values less than 0.001) and medium correlation to the liver function index aspartate aminotransferase and alanine aminotransferase (Spearman's correlations more than 0.63 and 0.57 with p-values less than 0.001). *Conclusions*: The study shows the statistics of the ratio of infiltrated portal boundary have correlation to the Ishak grade and liver function index. The proposed framework provides pathologists with a useful and reliable tool for hepatitis diagnosis.

## I. Introduction

Hepatitis (liver inflammation) may develop into cirrhosis or liver cancer in the late stage [1], and is thus associated with a large number of mortalities globally every year [2]. The early diagnosis and treatment of hepatitis is therefore of critical concern in reducing hepatitis-related deaths. In clinical practice, pathologists generally use the Ishak grading system [3] to estimate the progress of hepatitis based on a manual inspection of hematoxylin and eosin (H&E) stained slides of the liver. One component of the periportal interface hepatitis activity in the Ishak grading score is determined by the percentage of portal borders infiltrated by lymphocytes. Therefore, the accurate detection of lymphocyte-infiltrated periportal regions is of critical concern for hepatitis diagnosis.

**Fig. 1.**
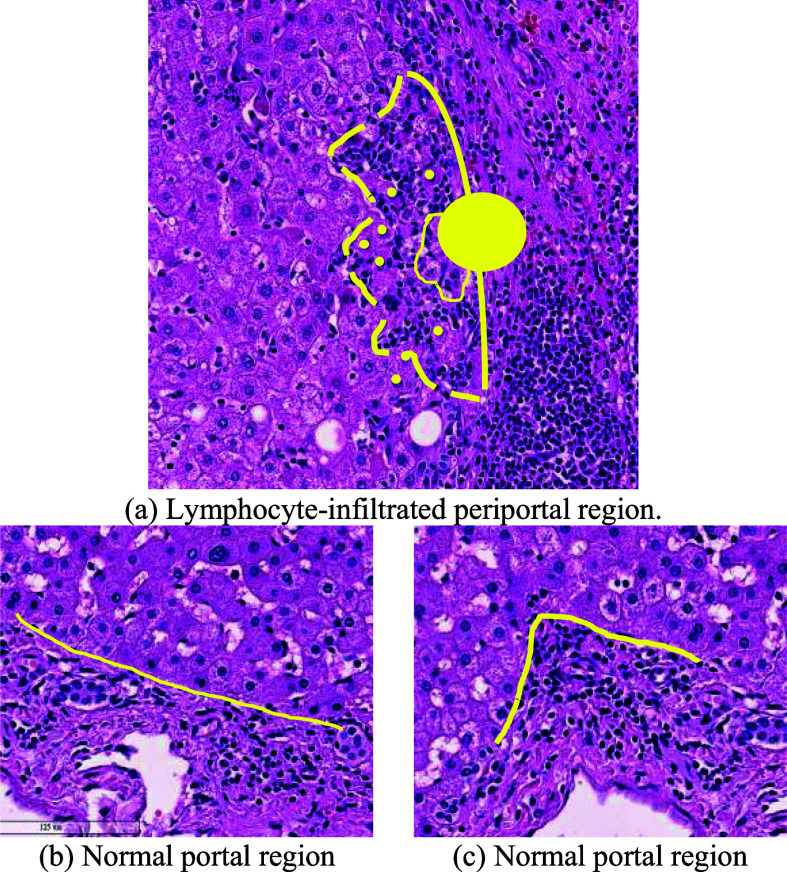
Lymphocyte-infiltrated periportal region and normal portal regions. The line segments are the original portal border, the circle and dots are the infiltrated hepatocytes, and the dashed line segment is the boundary of the infiltrated periportal region.

Deep learning models [4], [5], [6], [7], [8], [9] have achieved widespread success in the semantic segmentation of both natural images and medical images in recent years. However, identifying the infiltrated portal boundary regions in Whole Slide Images (WSIs) is extremely challenging. For example, the hepatic portal areas are very similar to the central vein areas, and are thus not easily distinguished using automated methods. Portal areas differ from central veins mainly in that they contain a small number of bile ducts. However, these ducts are present in only certain regions of the portal area, and may hence be missing in some patches of the WSI. This poses a major challenge to segmentation models in distinguishing portals and centrals at the pixel level using only neighboring pixels. Furthermore, in the infiltrated regions of the portal boundary, the lymphocytes break through the portal border and invade the nearby hepatic parenchyma regions, where they mix with the hepatocytes and generate ambiguous and highly-irregular portal boundaries, as shown in Fig. 1(a). The resulting mixture of lymphocytes and hepatocytes significantly complicates the task of identifying the portal boundary of the infiltrated regions.

To address these problems, the present study proposes a framework consisting of a Structurally-REfined Deep Portal Segmentation (SREDPS) module and an Infiltrated Periportal Region Detection (IPRD) module for detecting lymphocyte-infiltrated regions of the portal boundaries in liver WSIs.

**Fig. 2.**
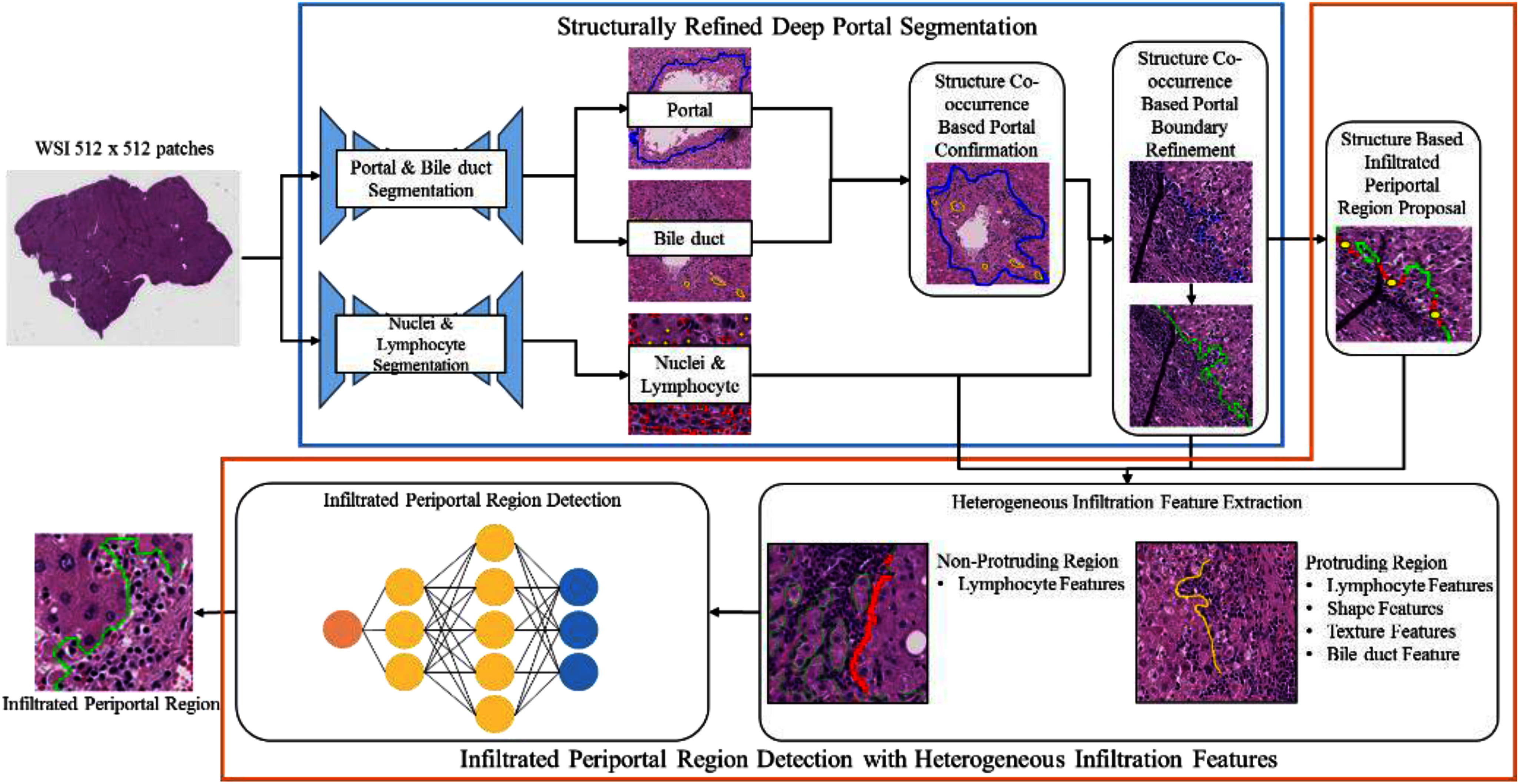
Flowchart of infiltrated periportal region detection framework.

As shown in Fig. 2, the SREDPS module consists of two structural analysis modules. A Structure Co-occurrence-based Portal Confirmation module is first used to distinguish between the central areas and portal areas in the WSI. As described above, one subtle difference between the two areas lies in the existence of bile duct structures in certain regions of the portal areas. Accordingly, having segmented the portal, bile duct, nuclei, and lymphocyte regions of the input WSI, the Structure Co-occurrence-based Portal Confirmation module checks for the co-occurrence of segmented portal regions and bile duct regions to confirm that the segmented portal regions are indeed portal areas, not central areas. Having detected the portal regions, a Structure Co-occurrence-based Portal Boundary Refinement module is employed to model the structural relationship between the portals and the lymphocytes and to refine the detected portal boundary such that it better fits the actual irregular boundary.

On completion of the SREDPS module, the IPRD module is used to detect the lymphocyte-infiltrated regions of the portal boundaries. A Structure-based Infiltrated Periportal Region Proposal module is first employed to identify possible infiltrated portal boundary candidates based on the structure and shape of the refined portal segmentations. A heterogeneous infiltration feature extraction process is then performed to extract multiple features from the candidate portal boundary regions. These features are input to an IPRD multilayer perceptron (MLP) to distinguish between normal periportal regions and lymphocyte-infiltrated periportal regions. The predictions of the IPRD model are then used to support either manual or automatic Ishak grading of periportal interface hepatitis for hepatitis diagnosis purposes.

## II. Materials and Methods

### A. Datasets

An H&E-stained liver WSI dataset was obtained from the Department of Pathology at National Cheng Kung University Hospital (NCKUH) in Taiwan. The dataset contained 100 WSIs with different periportal interface hepatitis Ishak grades, and was divided into a training set consisting of 43 WSIs and a testing set with 57 WSIs. All the WSIs were prepared with a 40X magnification using a Leica Aperio AT2 digital whole slide scanner. Table I shows the patient profiles of the training set and testing set. Fisher's exact test was performed on categorical variables and Mann-Whitney U test was applied on numerical variables including the three Ishak indices. The statistical analysis showed the two sets were drawn from the same distribution with high probability except the testing set has significantly more male patients compared to the training set. This study is approved by the Institutional Review Board of NCKUH and waived informed consent. The datasets generated during and/or analyzed during the current study are not publicly available, but are available from the corresponding author on reasonable request with approval of the Institutional Review Board.

**TABLE I:** Patient Profiles of the Training Set and Testing Set

Variables	**Training Set**	**Testing Set**	*p*
Age	62.30 (10.99)	61.40 (8.06)	0.375
Sex			0.001^**^
- Male	29 (67.44%)	53 (92.98%)	
- Female	14 (32.56%)	4 (7.02%)	
Hepatitis B Virus			0.569
- No	8 (18.60%)	8 (14.04%)	
- Yes	22 (51.16%)	33 (57.89%)	
- Missing	13 (30.23%)	16 (28.07%)	
Hepatitis C Virus			0.151
- No	13 (30.23%)	23 (40.35%)	
- Yes	18 (41.86%)	15 (26.32%)	
- Missing	12 (27.91%)	19 (33.33%)	
Hepatitis Virus (B+C)			0.501
- No	20 (46.51%)	25 (43.86%)	
- Yes	0 (0.00%)	2 (3.51%)	
- Missing	23 (53.49%)	30 (52.63%)	
AST	57.29 (28.98)	61.38 (61.12)	0.340
ALT	53.16 (39.23)	65.74 (69.18)	0.939
ALK-P	99.83 (51.96)	90.71 (38.11)	0.498
BIL-T	0.73 (0.29)	1.30 (3.05)	0.530
BIL-D	0.33 (0.16)	0.79 (2.84)	0.228
Ishak Periportal	0.95 (0.92)	1.05 (0.99)	0.665
Ishak Inflammation.	3.67 (2.12)	3.84 (2.10)	0.772
Ishak Fibrosis	4.40 (1.56)	4.48 (1.57)	0.804
Liver Fatty Change	10.22 (11.36)	11.06 (14.30)	0.796
Ishak Fib. ≥5			0.683
- No	20 (46.51%)	23 (40.35%)	
- Yes	23 (53.49%)	33 (57.89%)	
- Missing	0 (0.00%)	1 (1.75%)	
Cirrhosis, (Ishak Fib. 6)			> 0.999
- No	27 (62.79%)	35 (61.4%)	
- Yes	16 (37.21%)	21 (36.84%)	
- Missing	0 (0.00%)	1 (1.75%)	

1. Means, with standard deviations in parentheses, are presented for the numerical variables. Counts and percentages are presented for the categorical variables.

2. p-values for numerical and categorical variables are calculated using the nonparametric methods of Mann-Whitney U test and Fisher's exact test.

3. *p<0.05, **p<0.01, ***p<0.001

To train and evaluate the portal and bile duct segmentation model, 24 WSIs from the training set and 10 WSIs from the testing set were manually labeled. For each WSI, 3∼5 images with a size of 8192 x 8192 pixels were randomly sampled, giving a total of 125 images. Each image was cut into patches with a size of 512 x 512 pixels using a stride of 256 pixels. To train and evaluate the detection model for the infiltrated protruding boundaries, 2545 infiltrated boundary regions and 22771 non-infiltrated boundary regions were manually labeled in the training set, and 1905 infiltrated regions and 18847 non-infiltrated regions were labeled in the testing set.

### B. Structurally-Refined Deep Portal Segmentation (SREDPS)

To detect the lymphocyte-infiltrated periportal regions, it is first necessary to accurately segment the portal, hepatocyte and lymphocyte regions of the WSI. However, as described above, this is extremely challenging for automated methods since central areas often have a similar appearance to portal areas and are thus easily misidentified as such. Furthermore, the infiltrating lymphocytes often cause ambiguous and irregularly-shaped portal boundaries, which further complicate the segmentation task.

In the framework proposed in this study, these challenges are addressed through a Structurally-REfined Deep Portal Segmentation (SREDPS) module, which aims to reduce the ambiguity of the portal regions based on the structural relationships among the segmented regions of the portals, bile ducts, and lymphocytes, respectively. As shown in Fig. 2, the SREDPS module performs portal segmentation using a two-branch deep segmentation model, where the upper branch uses a DeepLabv3-based model [4] to perform portal and bile duct segmentation, and the lower branch uses the model proposed by Li et al. [10] to perform hepatocyte and lymphocyte segmentation.

In the SREDPS module, the ambiguity arising from the similarity between the appearance of the portal area and that of the central area is mitigated by examining the structural co-occurrence of the segmented portals and bile ducts. Furthermore, to resolve the problem of ambiguous and irregular portal boundaries caused by infiltrating lymphocytes, a structural co-occurrence evaluation of the portals and neighboring lymphocytes is also performed based on the predictions of the two segmentation branches. In particular, the lymphocytes infiltrating from the portal area to the hepatocyte region are detected, and a distance-based clustering algorithm (DBSCAN [11]) is used to cluster the lymphocytes around the portal area based on their spatial coordinates. Lymphocytes infiltrating from the portal area are then clustered together with those inside the portal area and used to refine the contour of the portal area accordingly.

Lymphocytes are usually scattered as tiny dotted regions in human tissue. Thus, to generate continuous portal region proposals, the Delaunay triangulation algorithm [12] is used to connect the infiltrating lymphocytes. In particular, triangles enclosed by nearby lymphocytes are considered to be portal regions and are retained, while those with edges longer than the minimum hepatocyte cell size (20 μm) are removed. The triangle regions which remain are then filled and merged with the original portal regions via a bitwise-OR operation to obtain the refined portal region boundary.

### C. Infiltrated Periportal Region Detection With Heterogeneous Infiltration Features

As described above, the infiltrated and non-infiltrated portal boundary regions are ambiguous and not easily distinguished. Thus, in the present study, candidate infiltrated periportal regions are first identified based on the structure of the portal regions, and the actual infiltrated regions are then detected from among these candidates based on an analysis of a given set of heterogeneous infiltration features.

#### 1) Infiltrated Periportal Region Proposal

**Fig. 3.**
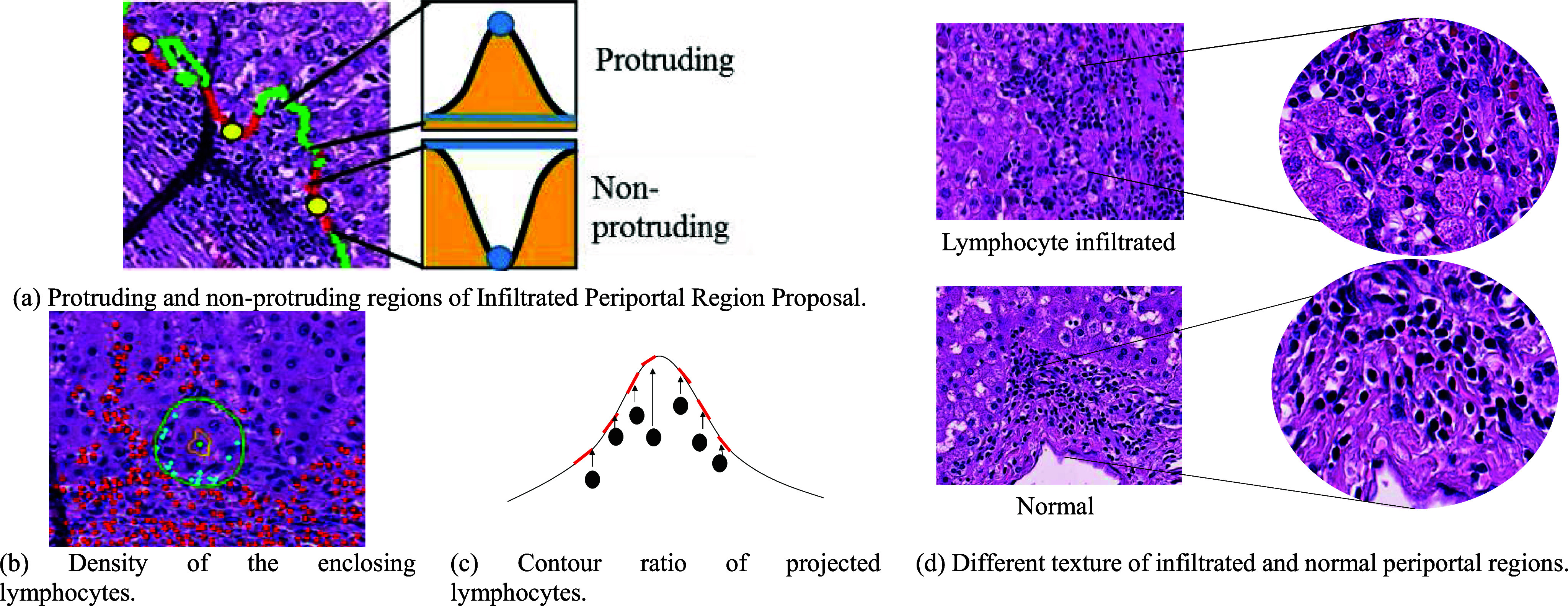
Infiltrated Periportal Region Proposal and Heterogeneous Infiltration Features.

Infiltrating lymphocytes often create protruding and highly-irregular regions on the portal boundary. Therefore, in the present study, candidate infiltrated portal regions are selected based on the structure of the portal boundary. In particular, for each point on the portal boundary, and the contour segment with a length equal to twice the hepatocyte diameter around it, a straight line is drawn between the two end points of the segment, as shown in Fig. 3(a). If the line passes through the portal area, the point is determined to be a protruding point; otherwise, it is taken to be a non-protruding point. The non-protruding segments of the portal boundary are constructed by concatenating all the continuous non-protruding points. The contour segments between the midpoints of two adjacent non-protruding segments are then determined to be protruding segments and are combined to form the protruding region of the boundary.

#### 2) Heterogeneous Infiltration Feature Extraction

For each candidate infiltrated periportal region, multiple heterogeneous features are extracted for further analysis purposes, where these features are specifically designed based on the characteristics of the infiltrated regions and their difference from normal regions, respectively. Twelve features are extracted for the protruding regions, and one feature is extracted for the non-protruding regions. As described in the following, the 12 features for the protruding regions include three lymphocyte features, four shape features, four texture features, and one bile ductule feature.

##### a) Lymphocyte Features

The protruding regions associated with lymphocyte infiltration are filled with lymphocytes. Therefore, the first feature is defined simply as the lymphocyte density in the protruding region. That is,

\begin{equation*}
{{f}_1} = \frac{{Number\ of\ lymphocytes\ }}{{Protruding\ region\ area}}\ . \tag{1}
\end{equation*}

Second, in lymphocyte-infiltrated regions, the hepatocytes are mixed with lymphocytes and are nearly enclosed by them. The density of the enclosing lymphocytes is thus taken as the second feature for the protruding region, as shown in Fig. 3(b). To identify these lymphocytes, the hepatocytes lying within a distance equal to the mean cell size (25 μm) from the portal boundary are first extracted, and a watershed algorithm [13] is applied to generate the cell regions. A dilation operation with a size of 25 μm is then applied to each cell region and all the neighboring lymphocytes are retrieved. If lymphocytes are found to exist in more than five out of the eight possible directions around a hepatocyte, the hepatocyte is considered to be enclosed by the lymphocytes. The density of the enclosing lymphocytes is then computed as follows:

\begin{align*}
& {{f}_2} =\\
& \frac{{Number\ of\ lymphocytes\ around\ infiltrated\ heaptocytes}}{{Protruding\ region\ area}}\ . \tag{2}
\end{align*}

In lymphocyte-infiltrated regions, the lymphocytes lie close to the boundary. Thus, the third lymphocyte feature is computed by projecting the lymphocytes in the protruding region onto the nearest portal boundary within a distance of 10 μm (i.e., a distance slightly longer than the average lymphocyte diameter (7 μm)) and then dividing the contour length of the projected lymphocytes by the contour length of the protruding region, as shown in Fig. 3(c). That is,

\begin{equation*}
{{f}_3} = \frac{{Contour\ length\ of\ projected\ lymphocytes}}{{Contour\ length\ of\ protruding\ region}}. \tag{3}
\end{equation*}

##### b) Shape Features

**Fig. 4.**
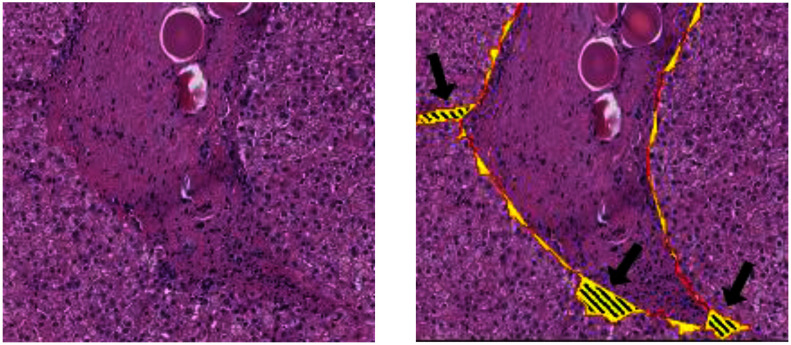
Portal tract branching or tangential cutting can cause protruding regions, but they are usually larger (shaded regions pointed by arrows). Thus, the ratios of the contour length to the portal perimeter length and area of the protruding region to the total area of the portal region are taken into consideration to reduce the ambiguity.

Four shape features are proposed to describe the shape of the protruding region, namely the steepness, mean curvature, contour length ratio, and protruding area ratio. The first feature, f_4_, describes the steepness of the protruding region (=(region h)/(region w)) and indicates the extent of the lymphocyte infiltration into the hepatocyte region. The second feature, f_5_, describes the mean curvature of the contour points on the protruding region and represents the irregularity of the protruding region. The protruding regions caused by natural shape variations of the portals, such as portal tract branching or tangential cutting, are usually larger than those produced by infiltrating lymphocytes (Fig. 4). Thus, the ratios of the contour length to the portal perimeter length and area of the protruding region to the total area of the portal region are taken as the third and fourth shape features, respectively. That is,

\begin{align*}
&{{f}_6} = \frac{{Contour\ length\ of\ protruding\ region}}{{Portal\ perimeter}}, \tag{4}\\
&{{f}_7} = \frac{{Area\ of\ protruding\ region}}{{Area\ of\ Portal\ area}}\ . \tag{5}
\end{align*}

##### c) Texture Features

Some non-infiltrated protruding regions are also filled with lymphocytes. The difference between such regions and the lymphocyte-infiltrated regions lies in the existence of fibrosis in the former case, as shown in Fig. 3(d). In the present study, the texture features of fibrosis are described using statistics of the corresponding Gray Level Co-occurrence Matrix (GLCM) [14]. In particular, eight GLCMs are created for each protruding region using two distances (3 μm and 5 μm) and four angles (0°, 45°, 90°, and 135°), respectively. For each GLCM, four statistics are determined, namely the contrast, dissimilarity, angular second moment, and energy. The statistics are then individually averaged over the eight GLCMs to obtain four texture features, namely $[ {{{f}_8},{{f}_9},{{f}_{10}},{{f}_{11}}} ]$.

##### d) Bile Ductule Feature

**Fig. 5.**
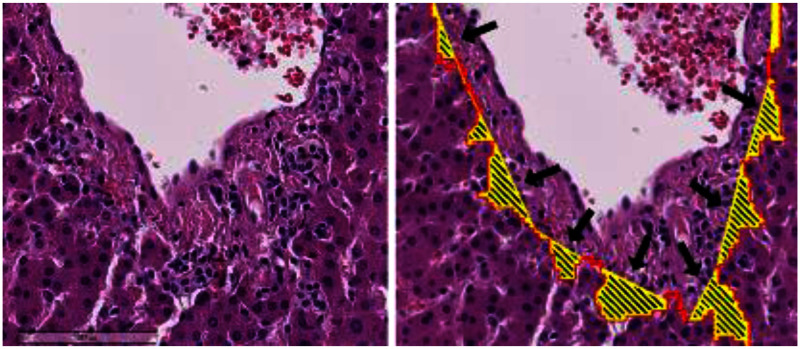
Periportal areas may contain bile ductules (left penal) causing protruding regions (shaded regions pointed by arrows in the right penal) but these regions are not true periportal infiltration. Therefore, bile ductule/protruding area ratio is taken into consideration to reduce the ambiguity.

Besides lymphocyte infiltration, periportal regions may also contain bile ductules causing protruding features mimicking interface activity (Fig. 5). Thus, the following bile ductule feature is computed based on the bile ductule area ratio:

\begin{equation*}
{{f}_{12}} = \frac{{Area\ of\ bile\ ductules}}{{Area\ of\ protruding\ region}}. \tag{6}
\end{equation*}

##### e) Lymphocyte Feature of Non-Protruding Region

Some infiltrated regions located between two infiltrated protruding regions may not be detected as protruding regions. Around these regions, most of the interior hepatocytes are enclosed by lymphocytes. Thus, the lymphocytes enclosing hepatocytes are first identified using the same method as that described above for feature f_2_. The lymphocytes are then projected to the nearest contour within a distance of 10 μm. Finally, the density of the projected enclosing lymphocytes is computed as

\begin{equation*}
{{f}_{13}} = \frac{{Number\ of\ projected\ enclosing\ lymphocytes\ }}{{Portal\ contour\ segment\ length}} \tag{7}
\end{equation*}

#### 3) Infiltrated Periportal Region Detection

The 12 heterogeneous features described above are used to train a multilayer perceptron (MLP) to distinguish between infiltrated protruding regions and natural protruding regions. The MLP consists of two layers with 16 and 8 hidden dimensions respectively. Since the two classes are imbalanced in the WSI dataset, the training process is performed using a weighted cross entropy (WCE) loss function with the form:

\begin{equation*}
- w*y\log \left( {\hat{y}} \right) - \left( {1 - y} \right)\log \left( {1 - \hat{y}} \right), \tag{8}
\end{equation*}

where w is the class weight, y is the ground truth, and $\hat{y}$ is the MLP prediction (i.e., infiltrated protruded region or natural protruding region).

For the case of the non-protruding regions, the regions are assumed to be infiltrated simply if any enclosing lymphocytes are projected onto the non-protruding region. That is, if ${{f}_{13}}$ of a non-protruding region is larger than one, it is considered an infiltrated region.

## III. Results

The IoU values for the portal and bile duct segmentation tasks reached 0.905 and 0.744, respectively. The performance of the IPRD MLP was evaluated region-wisely for each protruding region. The lymphocyte-infiltrated regions were taken as the positive class, and the non-lymphocyte-infiltrated regions were taken as the negative class. Table II shows the evaluation results obtained for different values of the class weight (w) in the WCE loss function. The optimal F1-score is 0.725 and is obtained using a weight of w = 3.

**TABLE II:** Performance of Infiltrated Periportal Region Detection for Different w

w	Specificity	Precision	Recall	F1-score
1	0.985	0.809	0.627	0.707
**3**	0.974	0.734	0.716	**0.725**
5	0.961	0.666	0.775	0.716
7	0.953	0.63	0.796	0.703
9	0.934	0.56	0.832	0.67

**Fig. 6.**
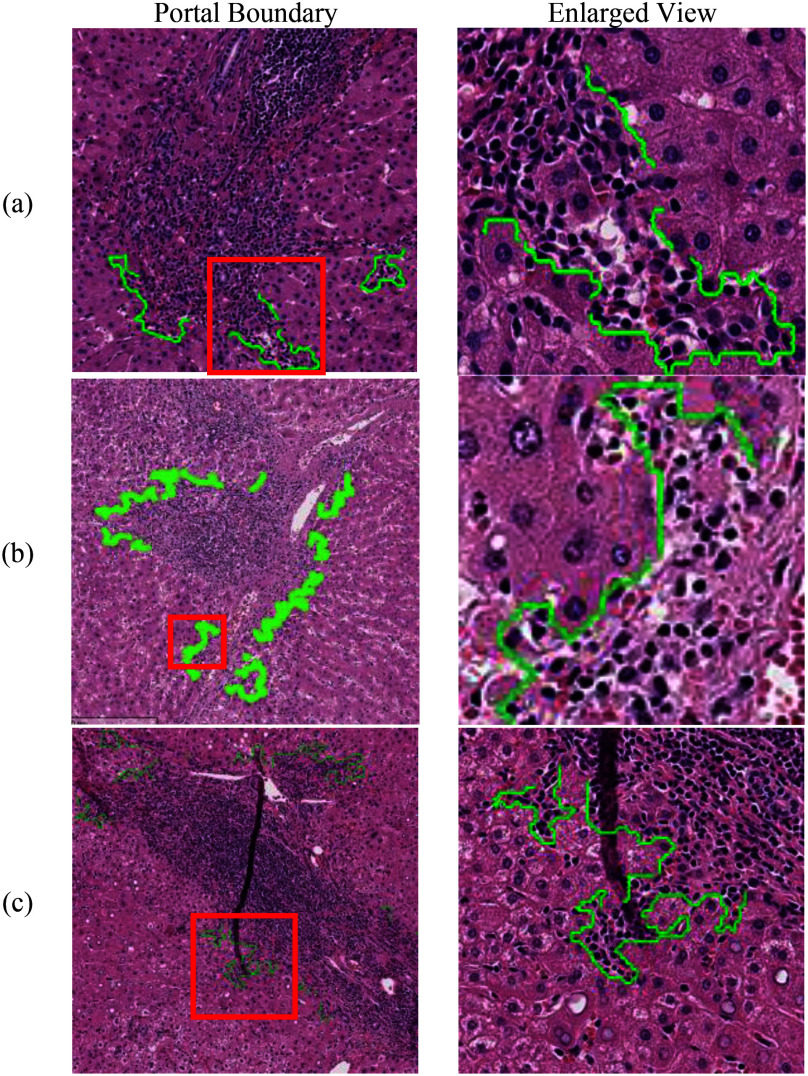
Typical detection results for infiltrated portal regions.

Fig. 6 shows the typical detection results obtained for the lymphocyte-infiltrated portal regions. Note that the images on the left-hand side of the figure show the entire portal area, while those on the right-hand side present magnified views of the portal regions within the red bounding boxes. In all of the images, the infiltrated portal boundary regions are marked in green. It is seen that in the infiltrated regions of the portal boundaries, the lymphocytes invade the nearby hepatic parenchyma regions, mix with the hepatocytes, and create highly-irregular portal boundaries. Nonetheless, the proposed method successfully detects these regions and achieves a precise fit between the predicted portal boundaries and the actual portal boundaries.

Based on the detected infiltrated regions of the portal boundary, the infiltrated portal boundary ratio was calculated for each portal in the WSI. The infiltrated ratio provides doctors with useful auxiliary information to evaluate the Ishak grade and diagnose hepatitis in clinical practice. To demonstrate this, an investigation was performed to evaluate the relationship between the calculated infiltrated portal boundary ratio and the patients’ different factors including age, sex, HBV, HCV, AST, ALT, as well as Ishak periportal interface hepatitis grading and total Ishak necroinflammatory score assigned to the WSI by consensus of two experienced pathologists (HWT and CWH). Five common statistics (the mode, median, average, mediant, and average of the highest 25%) were computed for the portal infiltrated ratios of all the portals in the WSI. The Spearman's correlations and p-values of the statistics with respect to the numerical factors were then evaluated. The Mann-Whitney U test were performed on categorical factors to the ratio statistics. Note that the mode is the most frequent number among the infiltrated ratios of all the portals in the WSI, while the median is the middle value that separates the higher and lower halves of the portal infiltrated ratio distribution. Finally, the average, mediant, and average of the highest 25% statistics are defined respectively as follows:

\begin{align*}
{{S}_{avg}} &= \frac{1}{n}\mathop \sum \limits_{portal}^{} \frac{{infiltrated\ boundary\ length}}{{portal\ perimeter}}, \tag{9}\\
{{S}_{mdt}} &= \frac{{\mathop \sum \nolimits_{portal}^{} infiltrated\ boundary\ length}}{{\mathop \sum \nolimits_{portal}^{} portal\ perimeter}}, \tag{10}\\
{{S}_{avg25}} &= \frac{1}{n}\mathop \sum \limits_{top25\% \ portal}^{} \frac{{infiltrated\ boundary\ length}}{{portal\ perimeter}}. \tag{11}
\end{align*}

**Fig. 7.**
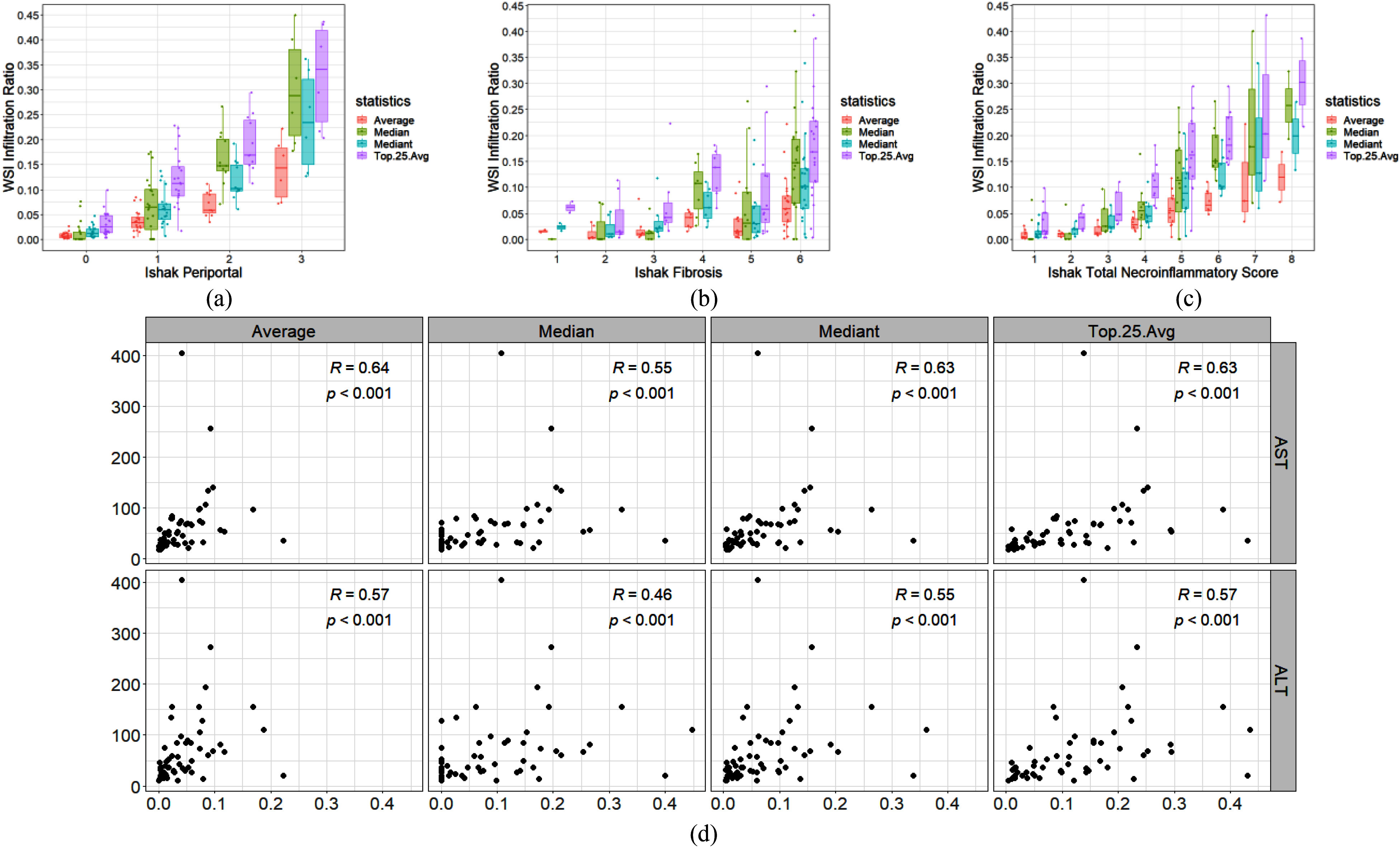
Distributions of the statistics of infiltrated boundary ratio in testing dataset. (a) Ishak periportal index to ratio statistics. (b) Ishak inflammation index to ratio statistics. (c) Ishak fibrosis index to ratio statistics. (d) AST and ALT to ratio statistics.

As shown in Fig. 7(a), four of the statistics were positively correlated with the interface hepatitis Ishak grade over the 57 WSIs in the testing set. The only exception was the mode statistic, which was zero in all cases because only a proportion of portals in the WSIs were infiltrated. Table III lists the Spearman's correlations and p-values as well as the p-values of the Mann-Whitney U test for the median, average, mediant, and average of the highest 25% statistics. The average, median, and average of the highest 25% statistics have high Spearman's correlations of over 0.87 and p-values of <0.001. These ratio statistics also have high correlation of over 0.83 and p-values of <0.001 to the total Ishak necroinflammatory score (Fig. 7(b)–(d)). In addition, the AST, ALT and Ishak fibrosis index also show medium correlation to the ratio statistics. In other words, the results confirm that the infiltrated portal boundary ratio detected by the proposed model is highly correlated with the Ishak grade of interface hepatitis, and thus provides doctors with a useful tool for diagnosis purposes.

**TABLE III:** Correlation Between Infiltrated Boundary Ratio Statistics and Patients’ Different Factors

**Factors**	**Median**	${\bm{p}}$	**Average**	${\bm{p}}$	**Mediant**	${\bm{p}}$	**Top 25 Average**	${\bm{p}}$
Age	-0.03	0.807	-0.11	0.398	-0.13	0.329	-0.13	0.340
Sex		0.668		0.522		0.482		0.563
HBV		0.638		0.402		0.500		0.402
HCV		0.035^**^		0.060		0.073		0.089
AST	0.55	< 0.001^***^	0.64	< 0.001^***^	0.63	< 0.001^***^	0.63	< 0.001^***^
ALT	0.47	< 0.001^***^	0.57	< 0.001^***^	0.55	< 0.001^***^	0.57	< 0.001^***^
ALK-P	0.24	0.137	0.18	0.250	0.20	0.210	0.17	0.288
BIL-T	0.12	0.379	0.09	0.507	0.10	0.480	0.12	0.399
BIL-D	0.02	0.898	-0.06	0.663	-0.04	0.766	-0.05	0.709
Ishak Periportal	0.82	< 0.001^***^	0.88	< 0.001^***^	0.87	< 0.001^***^	0.87	< 0.001^***^
Ishak Inflammation	0.78	< 0.001^***^	0.84	< 0.001^***^	0.84	< 0.001^***^	0.83	< 0.001^***^
Ishak Fibrosis	0.55	< 0.001^***^	0.53	< 0.001^***^	0.54	< 0.001^***^	0.51	< 0.001^***^
Liver Fatty Change	0.29	0.028*	0.29	0.033*	0.25	0.063	0.27	0.047*
Ishak Fib. ≥5		0.005^**^		0.005^**^		0.005^**^		0.008^**^
Cirrhosis, (Ishak Fib. 6)		< 0.001^***^		< 0.001^***^		< 0.001^***^		0.001^**^

1. *p<0.05, **p<0.01, ***p<0.001

2. The Spearman correlation coefficients and their corresponding p-values are used to assess the relationship between numerical factors and the infiltrated boundary ratio statistics.

3. p-values of infiltrated boundary ratio statistics of categorical factors are calculated using the Mann-Whitney U test.

## IV. Discussion

This paper has presented an SREDPS module for resolving the appearance ambiguity between central and portal areas in liver WSIs and for refining the highly-irregular portal boundaries in the lymphocyte-infiltrated regions. A structure-based infiltrated periportal region proposal module, together with an IPRD MLP trained on heterogeneous lymphocyte infiltration features, has additionally been proposed to reduce the ambiguity between infiltrated and non-infiltrated regions of the portal boundary. Based on the predictions of the IPRD model, the statistics of the ratio of the detected infiltrated portal boundary are highly correlated with the Ishak grade. Therefore, the proposed framework provides pathologists with a useful tool for evaluating periportal interface hepatitis component of the Ishak score in the diagnosis of hepatitis.

Some pathologic changes could cause periportal contour irregularity/protrusion mimicking interface activity, such as tangential cutting of branching portal tracts and ductular reaction. Therefore, to accurately identify the true interface activity, the proposed heterogeneous infiltration features contain both the negative features which against the existence of infiltrated regions and the positive features which support the infiltrated regions. For example, the ambiguity caused by the branching portal tracts can be reduced with the information of contour ratio (${{f}_6}$) and area ratio (${{f}_7}$). Similarly, the ductular reaction can be better identified from the infiltrated regions given the ductular area ratio (${{f}_{12}}$). Moreover, the lymphocyte feature ${{f}_2}$ defines an enclosed hepatocyte to be surrounded by lymphocytes in more than five out of the eight directions in periportal areas within a distance from the portal boundary. This feature helps to reduce the ambiguity caused by the sinusoidal lymphocytes that usually lying one sides of the hepatocytes or in the sinusoids of the lobules.

To the best of the authors’ knowledge, there is no other deep learning-based method specifically designed for detecting lymphocyte-infiltrated periportal regions or estimating the Ishak periportal interface hepatitis score. Periportal interface inflammation is important in diagnosis of chronic viral hepatitis and autoimmune hepatitis. Periportal interface inflammation, portal inflammation and lobular inflammation are scored separately in both Knodell and Ishak scoring systems [3], [15]. Previous studies to scoring inflammatory activities in liver by imaging analysis have been largely focused on lobular inflammation in nonalcoholic steatohepatitis (NASH) [16], [17], [18], [19]. Liu et al. has proposed a qFIBS automated technique for quantitation of fibrosis, inflammation, ballooning, and steatosis in NASH [17]. Although they quantified the inflammatory density separately in portal tract region, central vein region and perisinusoidal region, they did not measure the periportal interface inflammation. For comparison, the proposed method is compared with a WSI classification method DSMIL [20] and a segmentation method SegFormer [21]. DSMIL directly estimates the Ishak score from the WSIs. SegFormer segments infiltrated periportal regions. As the same to the propsoed method, the four statistics (the median, average, mediant, and average of the highest 25%) of the portal infiltrated ratios are calculated based on the infiltrated region prediction of SegFormer.

**Fig. 8.**
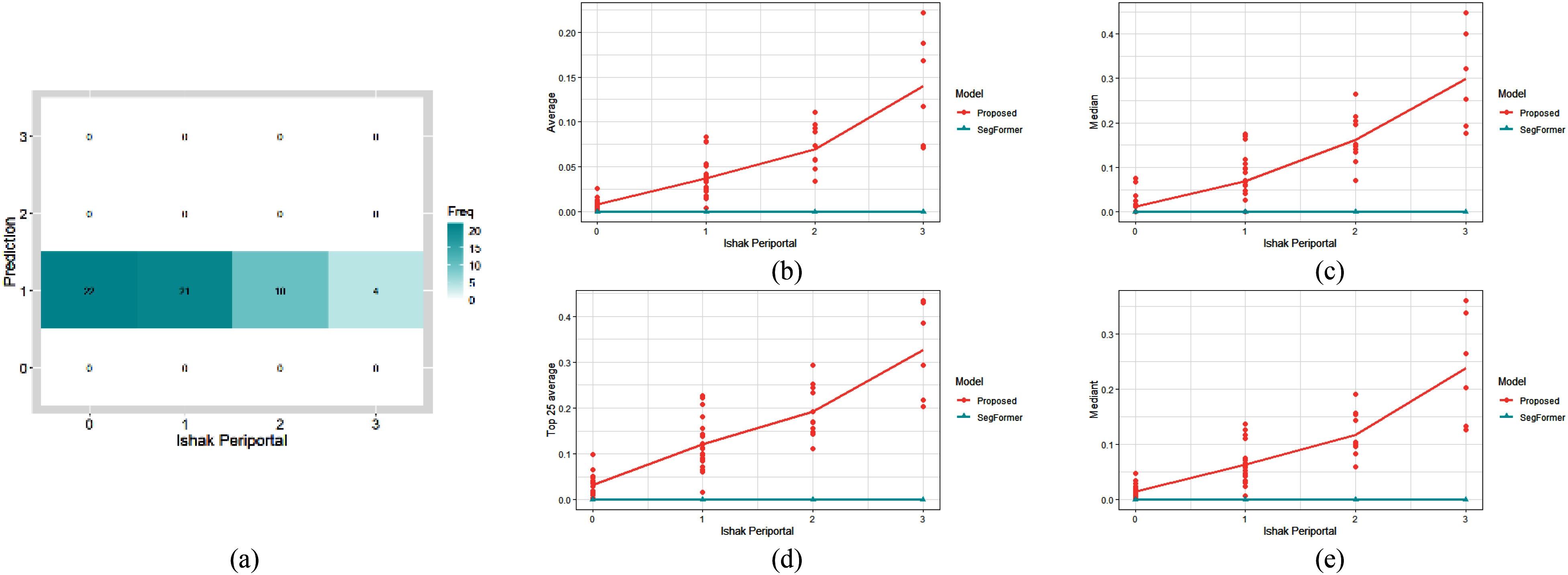
Analysis results of different methods. (a) Confusion matrix of DSMIL [20]. (b)--(e) The four statistics (the median, average, mediant, and average of the highest 25%) of the portal infiltrated ratios of all portals on the WSIs of SegFormer [21] and the proposed method.

Fig. 8 shows the results of the methods for comparison. Possibly because the infiltrating lymphocytes generate ambiguous and highly-irregular portal boundaries in the infiltrated regions, it is hard for models directly learn to detect lymphocyte-infiltrated periportal region or to estimate the Ishak periportal interface hepatitis score without additional guidance. Therefore, DSMIL [20] fails to distinguish different levels of Ishak periportal interface hepatitis and generate identical predictions for all levels of WSIs. SegFormer [21] also fails to identify infiltrated periportal regions and no infiltrated region is detected. On the other hand, the proposed method not only refines the portal boundary based on the structrual relations among the portals, bile ducts and cells, but also extracts heterogeneous infiltration features to represent the infiltrated periportal regions. Consequently, the proposed method can sucessfully detect infiltrated periportal regions. Moreover, the statistics of the infiltrated ratios of the portals also show high correlation to the doctors’ diagnosis. The experimental results show the effeictiveness of the SREDPS and the IPRD modules in the proposed method.

Periportal interface inflammation serves as direct evidence of liver injury in chronic hepatitis, including viral and autoimmune hepatitis. In clinical trials for chronic viral hepatitis treatment, the necroinflammatory score is frequently utilized to assess baseline histologic changes for inclusion criteria. It also aids in evaluating histologic improvement for therapeutic efficacy, forming a crucial part of the primary objective [22], [23]. Additionally, the score can function as a prognostic marker for disease relapse and guide treatment decisions. For instance, the presence of interface hepatitis upon drug withdrawal is associated with an 80% clinical relapse in autoimmune hepatitis [24], [25]. Histological evidence of interface hepatitis during corticosteroid treatment justifies the extension of the therapeutic period. In these clinical settings, an AI-based whole slide image (WSI) analysis framework can provide more objective and detailed information for disease evaluation, facilitating treatment decisions.

In the future, the presented study could be further extended in two directions. First, currently 13 heterogeneous lymphocyte infiltration features are computed to describe the possible infiltrated periportal regions. It takes an average of 20∼25 minutes to analyze a WSI. Feature selection policy could be adopted to identify and select critical features and reduce the computation time. Second, the Ishak periportal or periseptal interface hepatitis score is defined in 5 discrete levels, while different cases of the same Ishak score may have different severity of hepatitis and show different extent of lymphocyte infiltration. Based on the detected infiltrated periportal regions, artificial intelligence models could be developed to predict a continuous severity score and assist pathologists to better estimate the progress of hepatitis.

## V. Conclusion

The present study demonstrated the proposed AI-based WSI analysis framework could generate refined infiltrated portal boundary ratios that were significantly correlated with Ishak grade and liver function tests. The objective WSI infiltration ratios could not only assist pathologists in evaluation of interface hepatitis but also provide more detailed and sensitive information about hepatitis severity, disease progression, and treatment effects in clinical follow-up and future pharmaceutical development.

## VI. Conflict of Interest

No conflict of interest.

## VII. Author Contributions

Hung-Wen Tsai, Chien-Yu Chiou, Tsan-An Hsieh, Matthew M. Yeh, Meng-Ru Shen, and Pau-Choo Chung contributed to the conception and design of this study. Hung-Wen Tsai, Cheng-Yi Chen, Che-Wei Hsu, and Yih-Jyh Lin collected and annotated the pathological images. Tsan-An Hsieh developed and trained the infiltrated periportal detection model and conducted the experiments. Wei-Jong Yang, Min-En Hsieh conducted the experiments. Chin-Chun Chen performed statistical analysis of the experimental results. Hung-Wen Tsai, Chien-Yu Chiou, Wei-Jong Yang and Pau-Choo Chung prepared the manuscript. All the authors revised and approved the final version.
